# Cross-Domain Open Set Fault Diagnosis Based on Weighted Domain Adaptation with Double Classifiers

**DOI:** 10.3390/s23042137

**Published:** 2023-02-14

**Authors:** Huaqing Wang, Zhitao Xu, Xingwei Tong, Liuyang Song

**Affiliations:** 1 College of Mechanical Electrical Engineering, Beijing University of Chemical Technology, Beijing 100029, China; 2 Key Laboratory of Health Monitoring and Self-recovery for High-end Mechanical Equipment, Beijing University of Chemical Technology, Beijing 100029, China

**Keywords:** fault diagnosis, open set domain adaptation, transfer learning, rotating machinery, deep learning

## Abstract

The application of transfer learning in fault diagnosis has been developed in recent years. It can use existing data to solve the problem of fault recognition under different working conditions. Due to the complexity of the equipment and the openness of the working environment in industrial production, the status of the equipment is changeable, and the collected signals can have new fault classes. Therefore, the open set recognition ability of the transfer learning method is an urgent research direction. The existing transfer learning model can have a severe negative transfer problem when solving the open set problem, resulting in the aliasing of samples in the feature space and the inability to separate the unknown classes. To solve this problem, we propose a Weighted Domain Adaptation with Double Classifiers (WDADC) method. Specifically, WDADC designs the weighting module based on Jensen–Shannon divergence, which can evaluate the similarity between each sample in the target domain and each class in the source domain. Based on this similarity, a weighted loss is constructed to promote the positive transfer between shared classes in the two domains to realize the recognition of shared classes and the separation of unknown classes. In addition, the structure of double classifiers in WDADC can mitigate the overfitting of the model by maximizing the discrepancy, which helps extract the domain-invariant and class-separable features of the samples when the discrepancy between the two domains is large. The model’s performance is verified in several fault datasets of rotating machinery. The results show that the method is effective in open set fault diagnosis and superior to the common domain adaptation methods.

## 1. Introduction

In the modern industry, it is critical to keep the equipment running safely and stably [1]. As the integral parts of equipment, rotating components such as bearings and gears are prone to fault due to the harsh working environment, which will affect the stable operation of the equipment. Therefore, efficient fault diagnosis methods play an important role in early fault warning and maintenance [2], which can effectively reduce property losses and casualties caused by mechanical faults [3].

Traditional signal processing knowledge combined with machine learning methods, such as the BP neural network and Empirical Mode Decomposition (EMD), can effectively identify fault types and predict the operation status of components [4,5,6,7,8,9]. However, the feature extraction process of these methods seriously relies on professional knowledge and can easily consume many resources in the era of big data [10]. The emergence of Deep Learning (DL) can solve the problem of relying on the workforce [11]. The DL model represented by the Convolution Neural Network (CNN) and Long Short-Term Memory (LSTM) neural network is used in fault diagnosis, with promising results because of the powerful feature extraction capabilities [12,13,14,15,16,17,18]. Among most research, applying DL to a fault diagnosis requires two preconditions: (1) Test samples and samples participating in model training have the same label space. (2) There are enough labels in the training samples. However, due to the changes in the working environment of the equipment, the distribution of samples collected each time is different, and there are few samples with labels under the same working conditions. Therefore, it is important to realize fault diagnosis under different operating environments using labeled samples.

As a branch of the Transfer Learning (TL) method emerging in recent years, domain adaptation provides a new idea for cross-domain fault diagnosis using the concept of adversarial learning. It can use the existing and available label information to achieve a cross-domain fault diagnosis by reducing the distribution differences of the features [19,20]. In the current research on cross-domain fault diagnosis, it is mostly assumed that the two domains contain the same fault category. Under this assumption, the training model can extract the domain invariant features of the samples to achieve the purpose of cross-domain fault diagnosis.

**Figure 1:**
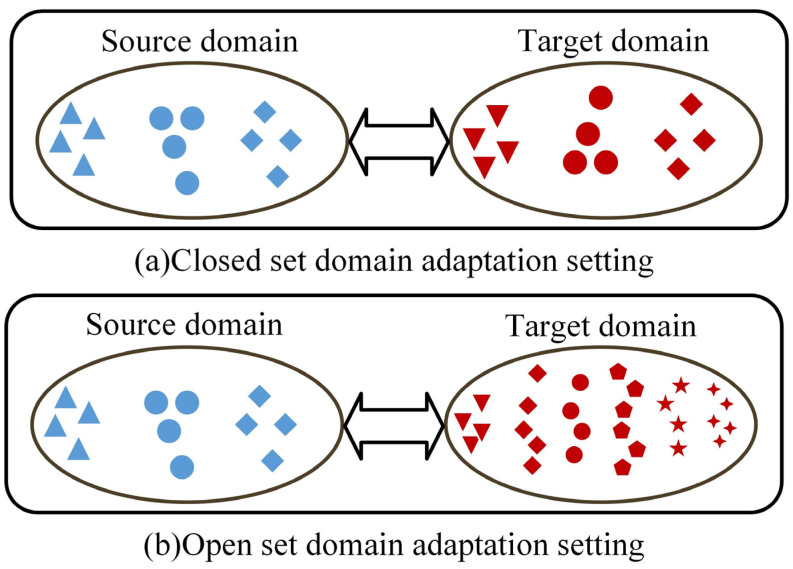
Difference between closed set and open set settings. (**a**) Closed set domain adaptation setting, (**b**) Open set domain adaptation setting.

However, the equipment status collected in a different environment is unknown, usually including classes outside the source domain, as shown in Figure 1. The existing domain adaptation model used in this case can lead to errors in sample alignment in the feature space and even affect the alignment between known classes under different conditions. In recent years, scholars have made initial achievements in the research of open set fault diagnosis, most of which are based on different theories to establish models that reject unknown samples [21]. However, these methods are prone to the problem of sample aliasing in the feature space when the discrepancy between the two domains is significant, resulting in low diagnostic accuracy.

**Figure 2:**
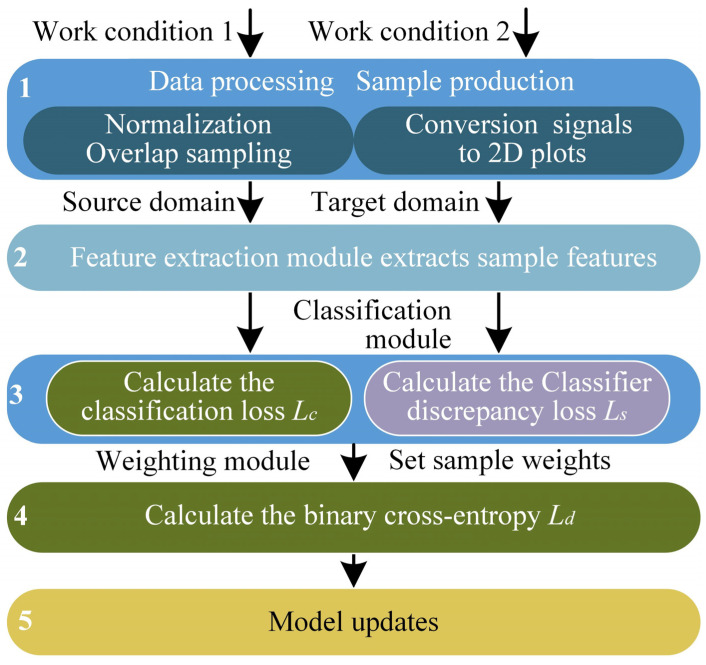
The framework of our method.

To solve the above problems and improve the accuracy of open set fault diagnosis, a method based on WDADC is proposed. The basic framework of the method is shown in Figure 2. The role of the feature extractor module is to extract the high-dimensional features of the processed data, the weighting module assigns weights to samples by calculating their similarity, and the classification module plays the role of correctly classifying the samples. Through the interaction between modules, we can accurately identify samples of the shared classes while separating samples of the non-shared class, thus achieving the goal of open set fault diagnosis.

The main contributions of this paper are as follows:

(1) For the problem of open set fault diagnosis, we design a weighting module based on Jensen–Shannon divergence in the adversarial model to evaluate the similarity of samples between the two domains. The target domain samples are assigned weights according to the similarity to facilitate alignment between shared class samples and separate the unknown samples.

(2) Considering the negative transfer problem in open set fault diagnosis, a weighted loss function is constructed to update the model in the direction of extracting domain invariant and class separable features of samples. When the distribution of samples in the two domains is quite different, the discrepancy between the double classifiers is used to improve the generalization of the model, and cross-domain open set fault diagnosis can be realized.

(3) The experiment on several mechanical fault datasets shows that the proposed method performs better than other domain adaptation methods.

The structural arrangement of this paper is shown as follows. Section 2 outlines the basic theory of the TL methods. Section 3 presents the proposed method in detail. Section 4 presents the designed validation experiments and analyzes the experimental results. Section 5 summarizes the entire paper and plans the future work.

## 2. Related Work

This section describes the application of TL in fault diagnosis. According to the different problems to be solved, we divided the application of TL in fault diagnosis into the closed set and open set. Additionally, according to the different modeling principles, we further distinguished between the methods. We reviewed each method mentioned, as shown in Table 1.

### 2.1. TL Methods Applied to Closed Set Fault Diagnosis

TL broadens the conditions for neural networks to be used in fault diagnosis. Its goal is to solve the problem that the characteristic information in signals under different working conditions is different and challenging to be recognized. According to the different technologies used, the development of TL in fault diagnosis can be divided into the following types: instance-based, mapping-based, model-based, and adversary-based.

The instance-based TL method assumes many overlaps in the features of the signal under different states. By weighting the source domain samples to construct a feature distribution similar to the target domain, the model has a good effect when testing the target domain samples after training [22,23].

The mapping-based TL method believes there will be some discrepancy between the two domains. Still, the discrepancy can be eliminated through different mapping methods in the feature space so that similar samples can be gathered together. Different samples can be separated from each other to achieve the goal of cross-domain fault diagnosis [24,25].

The model-based TL considers that the shallow part of the network model is less relevant to the final classification task, and it usually extracts the macro features of the fault. The labeled samples can be used to train the shallow part of the network model. The output layer parameters are adjusted by fine-tuning to accurately classify the target domain samples [26,27].

The idea of adversary-based TL comes from adversarial learning. Through the training objectives of each module in the model, the features of the samples extracted in the model can be separated by the classifier without being distinguished by the domain discriminator [28]. As the representative of the adversarial model, the domain adaptation model expects that the domain invariant and class separable features can be extracted after training. This method has been widely studied in cross-domain fault diagnosis [29,30,31].

TL has made good research progress in cross-domain fault diagnosis. Still, the above methods often default that the data in two domains have the same class, which limits the practical development of TL in fault diagnosis.

### 2.2. TL Methods Applied to Open Set Fault Diagnosis

In industrial practice, acquiring fault labels will consume a lot of financial and human resources. Due to changes in operation and environment, the classes of signals collected in the equipment are unknown. The use of common TL methods will cause serious aliasing between non-shared class and shared class samples during feature alignment. Therefore, cross-domain open set fault diagnosis has become an urgent development direction. The existing open set fault diagnosis methods are mainly considered from the perspective of modeling, which is primarily divided into discriminative and generative models [38].

The discriminative models in open set fault diagnosis include the traditional machine learning-based and deep neural network-based models. The machine learning-based model establishes a mechanism to reject non-shared samples by setting an empirical threshold of the machine learning model or analyzing the distribution of abnormal data in combination with extreme value theory (EVT) to achieve open set fault diagnosis [32,33]. However, this method has the problem that, once samples are recognized as unknown classes, they cannot be correctly classified again through training iterations. The deep neural network-based model mainly uses the powerful feature extraction capability of DL and solves the inherent closed set problem caused by the normalization of the Softmax layer in the network [34,35] to achieve the goal of open set fault diagnosis by identifying samples of non-shared classes. However, the negative transfer problem caused by the misjudgment of these methods is the urgent research direction.

The application of generative models in open set fault diagnosis is achieved through adversarial learning. The generation model generates samples with similar characteristics to the actual samples by learning the sample characteristics to facilitate the model’s training and increase the robustness of the model, which can also solve the data imbalance and small sample problems. Non-instance-based generative models combined with EVT or the empirical threshold setting can achieve open set fault diagnosis through the adversarial training of modules [36,37]. This method relies heavily on the validity of the generated samples, especially the generation of unknown samples needed to strengthen the stability and reliability further.

## 3. Proposed Method

Unlike models such as the Domain Adaptation Neural Network (DANN) applied to a closed set domain adaptation problem, the Open Set Domain Adaptation by Backpropagation (OSBP) [39] aims to correctly identify the shared class samples in the target domain and classify the non-shared class samples as unknown classes.

### 3.1. Problem Description

In open set fault diagnosis, the datasets with label information are defined as the source domain, while the target domain are the datasets without a label. $\left\{x_{i}^{s},y_{i}^{s}\right\}$ and $\left\{x_{i}^{t},y_{i}^{t}\right\}$ are the *i*-th sample and its label in the source and target domains. In the open set problem, the labels of the source domain are a subset of the labels of the target domain, the intersection of the two is called the shared class, and the labels are set as *M* classes, according to the number of sample classes. In the target domain, the complementary set of the source domain is called the non-shared class, and the label is set to *M* + 1.

### 3.2. OSBP

In OSBP, the model mainly includes a feature extractor and a classifier. The feature extractor maps the samples to the same feature space. The classifier receives the features output from the feature extractor and outputs the (*M* + 1)-dimensional probability through the Softmax function. The OSBP forms an adversarial relationship between the feature extractor and the classifier by the Gradient Reversal Layer (GRL) to train the model. Through continuous training, the feature extractor is optimized to maximize the loss of the classification, which helps to align the samples between the two domains. The probability $p_{x^{t}}^{M+1}$ that the sample is in the non-shared class is compared with the set threshold *t* by the output of the classifier. When *p < t*, the samples are classified as shared classes, and otherwise, they are classified as non-shared classes. This method aims to construct a decision boundary between shared class samples and non-shared class samples when the label of the target domain sample is unknown. The training objectives of the method are shown as follows:

(1) $$\begin{array}{l} \underset{G}{\min}L_{s}-L_{t}\\ \underset{C}{\min}L_{s}+L_{t} \end{array}$$

where *L_s_* is the classification loss, and *L_t_* is the binary cross-entropy loss of the sample classification result concerning the threshold *t*, with *t* standing at 0.5. From the training objective, it can be seen that the feature extractor expects to maximize the loss of the classification so that the probability of a sample being classified as a non-shared class is far from *t*. On the other hand, the classifier expects the probability of a sample being classified as a non-shared class to converge to *t*. The model parameters are continuously updated by the backpropagation of loss.

### 3.3. Proposed Method

**Figure 3:**
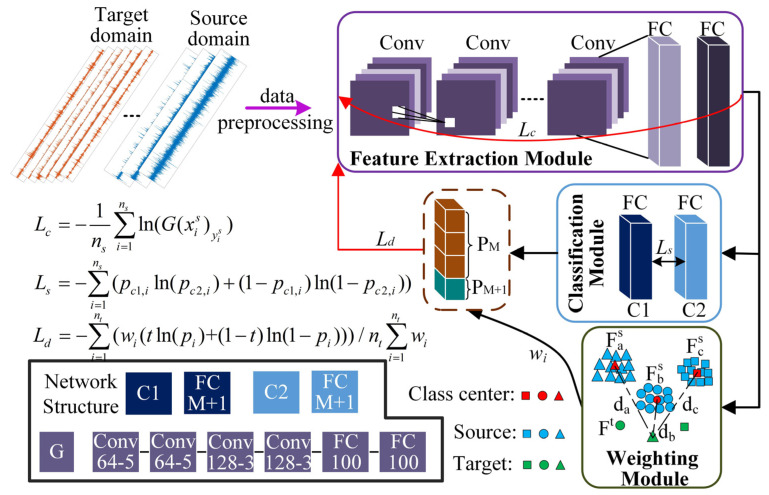
The training process and model structure of the proposed method.

In OSBP, due to non-shared class samples, the corresponding samples in the two domains are prone to aliasing during feature alignment, resulting in a negative transfer. Therefore, in WDADC, a weighting module is designed to improve the OSBP. By measuring the distance of the sample in the feature space, the samples are assigned different weights, thus promoting the positive transfer between shared classes in the two domains. In addition, using the discrepancy between different classifiers, the generalization of the proposed method is improved to achieve an excellent cross-domain open set diagnostic performance of the model. The training process and model structure of this method are shown in Figure 3.

(1) Feature extraction module: the module is denoted by *G* and consists of four layers of convolution and two layers of full connection, where each convolutional is connected with a batch normalization (BN) layer afterward. This module maps the samples to the same feature space and extracts the high-dimensional features. The features of the samples are output when going through the feature extraction module, as shown in Formula (2):

(2) $$\begin{array}{l} F_{i}^{t}=G(x_{i}^{t})\\ F_{i}^{s}=G(x_{i}^{s}) \end{array}$$

where $F_{i}^{s}$ and $F_{i}^{t}$ are the high-dimensional features of the *i*-th source domain and target domain samples.

For the labeled samples, the cross-entropy loss training model is calculated by the label information to correctly diagnose the fault, and the loss is shown in Formula (3):

(3) $$L_{C}=\frac{1}{n_{s}}\sum_{i=1}^{n_{s}}L_{CE}(F_{i}^{s},y_{i}^{s})$$

where *n_s_* is the number of labeled samples, and *L_CE_* is the cross-entropy loss.

(2) Weighting module: The module is denoted by *W*, and the novelty of this paper is that we design a weight calculation method based on Jensen–Shannon (JS) divergence, which assigns different weights to the samples by calculating the distances between the samples of the two domains in the feature space to promote a positive transfer between the shared classes. First, the class centers of the labeled samples in the feature space are calculated as follows:

(4) $$C_{a}=\frac{1}{n_{a}}\sum_{i=1}^{n_{a}}F_{a,i}^{s}$$

where *C_a_* is the class center of the *a*-th class, *a* ∈ {1, 2, …, M}, *n_a_* is the number of *a*-th class samples, and $F_{a,i}^{s}$ is the feature of the *a*-th class samples in the source domain.

Due to the existence of non-shared classes and the differences in working conditions, the feature distribution between the two domains is not precisely the same. The JS divergence is highly sensitive to the differences between two distributions and has a good diagnostic performance in different degrees of fault [40], and as a variation of Kullback–Leibler (KL) divergence, the JS divergence makes improvements over the symmetry and value domain range, making it more accurate in similarity discriminations. Therefore, to calculate the similarity between the sample and the class center in the two domains, JS divergence is used to calculate the method of their differences. In this paper, the distance between the sample features and the class centers in the two domains is calculated as follows:

(5) $$D_{JS,a}(C_{a}|F_{i}^{t})=\frac{1}{2}D_{KL}(C_{a}|\frac{C_{a}+F_{i}^{t}}{2})+\frac{1}{2}D_{KL}(F_{i}^{t}|\frac{C_{a}+F_{i}^{t}}{2})$$

where $D_{JS,a}(C_{a}|F_{i}^{t})$ is the JS divergence between the *i*-th sample feature in the target domain and the *a*-th class sample center source domain. *D_KL_* represents the *KL* divergence, which is calculated as shown in Formula (6):

(6) $$D_{KL}(M|N)=\sum_{i=1}^{v}M_{i}\ln(\frac{M_{i}}{N_{i}})$$

where *v* denotes the dimension of the output of the *G* module, and *M_i_* and *N_i_* represent the *i*-th elements of the vector.

The distance between the sample and each class center in the feature space is calculated, and the sum of the distances is used as the similarity judging index between the *i*-th samples in the two domains, as shown in Formula (7):

(7) $$D_{i}=\sum_{a=1}^{M}D_{JS,a}(C_{a}|F_{i}^{t})$$

When *D_i_* is smaller, we consider that this target domain sample is more similar to the source domain. Therefore, *D_i_* is normalized to [0, 1], and the difference between 1 and it is taken as the weights of this target domain sample in the model, as shown in Formula (8):

(8) $$w_{i}=1-\frac{D_{i}-D_{\max}}{D_{\max}-D_{\min}}$$

where *w_i_* is the weight generated by the weighting module for the *i*-th sample in the target domain.

(4) Classification module: The classification module is denoted by *C*, including two independent classifiers *C*1 and *C*2, both consisting of a fully connected layer and a BN layer. This module receives high-dimensional features from the feature extraction module and classifies them into *M* + 1 classes. The loss for each target domain sample is calculated by binary cross-entropy, as shown in Formula (9):

(9) $$L_{t}^{i}=-t\ln(p_{x_{i}^{t}}^{M+1})-(1-t)\ln(1-p_{x_{i}^{t}}^{M+1})$$

where $L_{t}^{i}$ is the binary cross-entropy loss of the *i*-th target domain sample, and $p_{x_{i}^{t}}^{M+1}$ is the probability that the *i*-th sample is recognized as a non-shared class.

The weights obtained from the calculations are added to the optimization process to obtain the weighted binary cross-entropy loss:

(10) $$L_{d}=(\sum_{i=1}^{n_{t}}L_{t}^{i}w_{i})/(n_{t}\sum_{i=1}^{n_{t}}w_{i})$$

where *n_t_* is the number of samples in the target domain, and *L_d_* is the total loss.

There is only one classifier in most models, which is prone to overfitting, resulting in low efficiency in the cross-domain fault diagnosis. In this paper, we use the discrepancy between double classifiers, generated by the complexity of the model and the initialization of the parameters, to promote the alignment between cross-domain samples by maximizing this discrepancy. It can improve the generalization and stability of the model. The calculation process is as shown in Formula (11):

(11) $$L_{s}=-\sum_{i=1}^{n_{s}}(p_{c1,i}\ln(p_{c2,i})+(1-p_{c1,i})\ln(1-p_{c2,i}))$$

The training objectives of this method are shown as follows. The adversarial relationship between classification and feature extraction modules is formed through GRL to achieve a continuous and stable diagnosis.

(12) $$\begin{array}{l} \underset{G}{\min}L_{c}-L_{d}+L_{s}\\ \underset{C}{\min}L_{c}+L_{d}-L_{s} \end{array}$$

## 4. Experimental Methods

In this section, we designed experiments on multiple datasets of a mechanical fault to verify the proposed method.

### 4.1. Datasets Description

(1) Laboratory Gearbox Dataset

**Figure 4:**
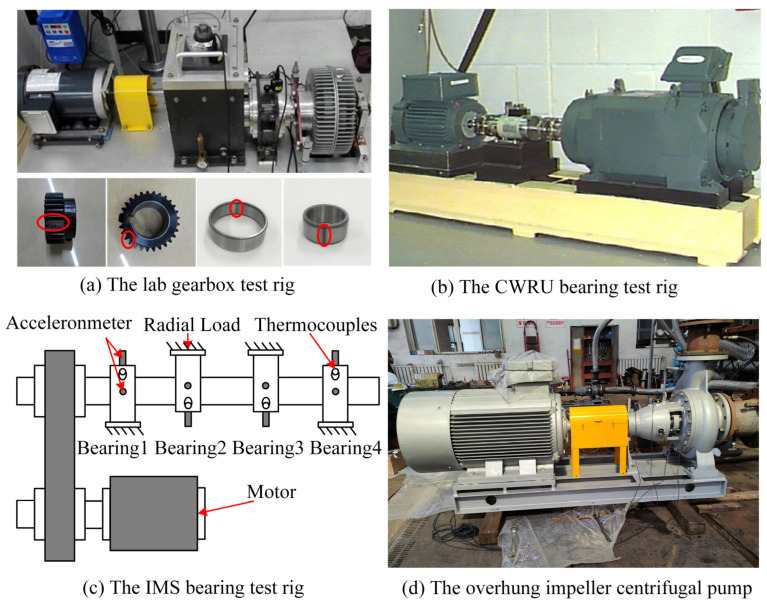
The test rigs. (**a**) The lab gearbox test rig, (**b**) The CWRU bearing test rig, (**c**) The IMS bearing test rig, (**d**) The overhung impeller centrifugal pump.

As shown in Figure 4a, the laboratory gearbox test rig consists of acceleration sensors, a braking system, and a gearbox. The state of the gear is divided into the chipped tooth, root wear, and healthy conditions, and the state of the bearing is divided into the inner race fault and outer race fault. The sampling frequency is set to 100 kHz, and the different working conditions are set by adjusting the rotational speed. Six types of status data are collected under 1200 rpm, 1500 rpm, and 1800 rpm bearing an inner race and gear root wear compound fault (IR), bearing an inner race and gear-chipped tooth compound fault (IT), bearing an inner race fault (I), bearing an outer race and gear root wear compound fault (OR), bearing an outer race and gear chipped tooth compound fault (OT), and bearing an outer race fault (O).

(2) The CWRU Dataset

As shown in Figure 4b, the Case Western Reserve University (CWRU) test rig consists of sensors, a motor, and an electronic controller. The bearing damage is a single-point damage by EDM. The bearing data of its drive end is used for testing. Set the sampling frequency to 12 kHz and different working conditions by adjusting the load. Four types of status data are collected under 0 hp, 1 hp, and 2 hp loads: inner race fault, rolling fault, outer race fault, and healthy conditions.

(3) The IMS dataset

As shown in Figure 4c, the Intelligent Maintenance Systems (IMS) test rig consists of accelerometers, a motor, bearings, and thermocouples. By applying a longitudinal load to the bearing, the whole process of bearing from healthy conditions to a fault is recorded. Set the sampling frequency to 20 kHz, and the speed is 2000 rpm. Four status data are collected, including the inner race fault, rolling fault, outer race fault, and healthy conditions.

(4) The centrifugal pump dataset

As shown in Figure 4d, the overhung impeller centrifugal pump dataset is collected in the industrial scene. The sampling frequency is set to 32.8 kHz, and the data of bearing healthy conditions, inner race fault, outer race fault, and rolling fault are collected under 745 rpm and 1485 rpm. The signal contains more interference components than the data collected in the laboratory and the public dataset.

### 4.2. Experiment Settings for Transfer Tasks

#### 4.2.1. The Transfer Tasks between the Same Equipment

For the laboratory gearbox dataset, to avoid the deviation of the experimental results caused by the fault types, the shared and non-shared classes are switched when different transfer tasks are set according to the rotational speed, as shown in Table 2. The labels are set to 0, 1, and 2, according to the order in the shared class in the table. The remaining data are the non-shared part, and the label is set to 3.

For the bearing dataset of CWRU, the inner race fault, rolling fault, and healthy conditions data are set as the shared class, and the labels are set as 0, 1, and 2 in turn. The outer race fault data is the non-shared part, and the label is set as 3. The tasks according to the load settings are shown in Table 3.

In the laboratory gearbox dataset experiments, the source domain contains 4500 samples, 1500 of each class. In the target domain, the number of samples per class in the shared part is 1000 and 500 samples per class in the non-shared part, totaling 4500, and the samples in the shared class in the target domain account for 66.67% of the total samples. In the experiments on the bearing dataset of CWRU, the source domain contains 3000 samples, 750 of each class, and 750 samples per class in the target domain, totaling 3000, and the samples in the shared class account for 75% of the total samples.

#### 4.2.2. The Transfer Tasks between the Different Equipment

For the conditions of different equipment, the CWRU and the IMS datasets are used to verify the diagnostic effect of the proposed model. It can be found from the introduction that the test rigs for collecting the two datasets are different in terms of load, speed, etc. Select the IMS dataset and the CWRU dataset with a 3 hp load to set the transfer tasks. The shared classes are the rolling fault, outer race fault, and health conditions. Set the labels to 0, 1, and 2 in order. Set the inner race fault to only exist in the target domain, and set the label to 3. The two domains, respectively, contain 3000 samples, including 1000 samples of each class in the source domain and 750 samples of each class in the target domain.

### 4.3. Data Preprocessing

At present, most of the samples for the fault diagnosis of equipment use time domain vibration signals, or arrange time domain one-dimensional vibration signals into matrices and convert them into two-dimensional images. However, due to the complex work environment of the equipment, the collected vibration signal is usually greatly interfered with by external vibration sources, which is easy to show as nonstationary, and a single time domain cannot fully express the relationship between the collected signal and the fault. Therefore, in this paper, the time–frequency image jointly represented by the time domain and frequency domain is selected as the input of the model, which can contain more fault information.

The common time–frequency analysis includes short-time Fourier transform (STFT) and a wavelet analysis. One of the distinctions between the two methods is the basis function. The basis function of STFT is a sine signal. The original signal is constructed by the superposition of sine signals of different frequencies. In the wavelet analysis, the basis function has a lot of selectivity and can perform scale transformation, which can effectively avoid the problem of time domain resolution in STFT.

In all the transfer tasks in this paper, we transform the time domain vibration signal into time–frequency images through a wavelet analysis. First, the collected vibration signals are normalized to [0, 1] to reduce the impact of the abnormal values. Then, the overlapping sampling is carried out when the overlapping amount is 600, and the length of each sample is 1024. After the time–frequency image of the one-dimensional vibration signal is generated by the wavelet analysis, the time–frequency image is grayed to reduce the redundant information in the sample and speed up the calculation efficiency of the model.

For the model parameters, according to the experience of intelligent diagnosis, the number of epochs is set to 400, the batch size is 16, the model is optimized using Stochastic Gradient Descent (SGD), the momentum is 0.9, the learning rate is 0.001, and the sample input size is 3 ∗ 32.

### 4.4. Competitors

To prove the performance of the proposed model, some domain adaptation methods are used for experimental comparison.

(1) Deep correlation alignment (CORAL): This method reduces the difference in feature distribution by aligning the statistical features of the two domains in the feature space.

(2) DANN: As a typical domain adaptation neural network, this method correctly recognizes test samples through continuous adversaries between the feature extractor and domain classifier in training.

(3) OSBP: As the base model before improvement, this method has the function of separating the non-shared samples while the shared samples are recognized in the feature sample space.

(4) OpenMax: As a typical method to solve the open set problem, it uses the EVT to fit the Weibull distribution with known samples to build a model that can reject unknown samples.

(5) A new adversarial network with multiple auxiliary classifiers (ANMAC): This method uses multiple auxiliary classifiers to evaluate the weight of each sample and sets a soft threshold to establish a model for open set recognition [21].

### 4.5. Experimental Results and Analysis of the Same Equipment

#### 4.5.1. Experimental Results

**Figure 5:**
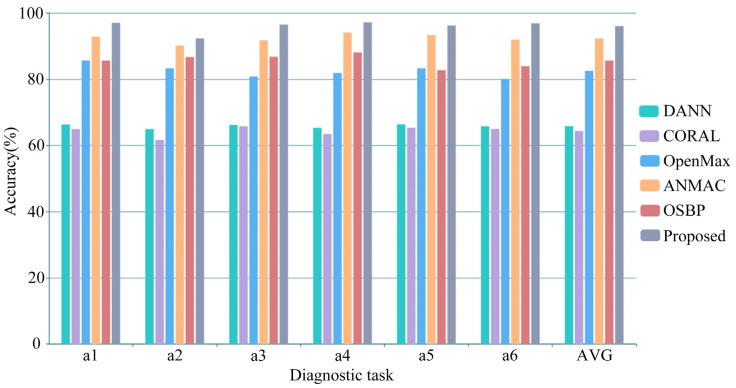
Diagnosis performance comparisons in the lab gearbox dataset.

The diagnostic results of the laboratory gearbox dataset diagnostic task are shown in Table 4 and Figure 5. The average accuracy of DANN, CORAL, OpenMax, ANMAC, OSBP, and the proposed method is 65.81%, 64.34%, 82.49%, 92.34%, 85.62%, and 96.01%, respectively. The proposed method is superior to the comparison methods in all diagnostic tasks. Specifically, the average accuracy of the traditional domain adaptation models DANN and CORAL is about 65%, which is close to the proportion of shared class samples in the target domain. It can be predicted that the two methods cannot separate unknown class samples. For OpenMax, using EVT can separate some unknown class samples, but the domain adaptation problem cannot be solved well, resulting in a low accuracy. ANMAC achieved more than 90% accuracy in all diagnostic tasks, reducing the side effects of the fixed threshold in OSBP, and dramatically improved the accuracy compared with OSBP. However, the volatility of the soft threshold can reduce the model’s performance, making the accuracy unable to be further enhanced. The proposed method assigns weights to the samples, promoting a positive transfer between shared class samples and keeping non-shared class samples away by calculating the similarity, solving the problem of cross-domain open set fault diagnosis.

**Figure 6:**
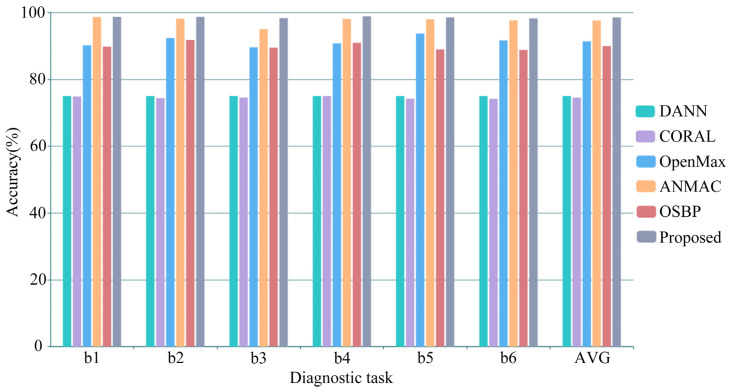
The diagnosis performance comparisons in the CWRU dataset.

The classification results of the diagnostic tasks in the CWRU dataset are shown in Table 5 and Figure 6. The average accuracy of DANN, CORAL, OpenMax, ANMAC, OSBP, and the proposed method is 75.00%, 74.51%, 91.35%, 97.58%, 89.94%, and 98.53%, respectively. Similar to the diagnosis results in the laboratory gearbox dataset, the accuracy of DANN and CORAL is close to the proportion of shared samples in the target domain, and they cannot play a role in the open set fault diagnosis. Due to the apparent fault features of the signals and the slight differences under different working conditions in this dataset, the accuracy of OpenMax was dramatically improved, reaching more than 90%. Similarly, the reduction of the fluctuation of the soft threshold also improved the accuracy of ANMAC. The proposed method is superior to the comparison methods in all diagnostic tasks, and the low standard deviation proves that it has good diagnostic stability.

#### 4.5.2. Feature Visualization and Confusion Matrix

**Figure 7:**
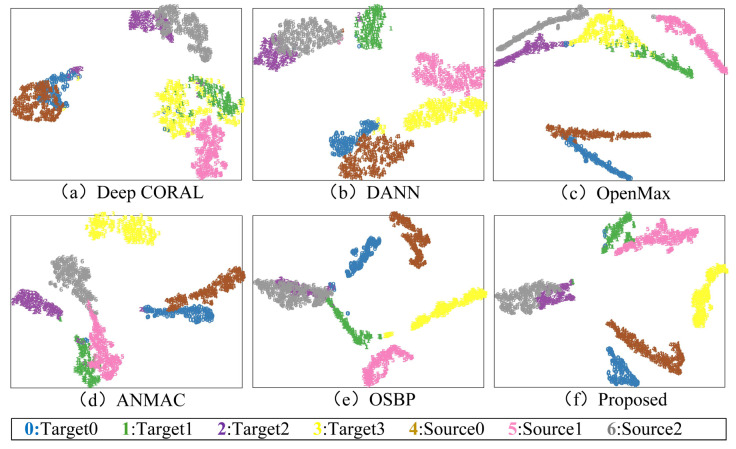
Feature visualization for transfer task a1. (**a**) Deep CORAL, (**b**) DANN, (**c**) OpenMax, (**d**) ANMAC, (**e**) OSBP, (**f**) Proposed.

**Figure 8:**
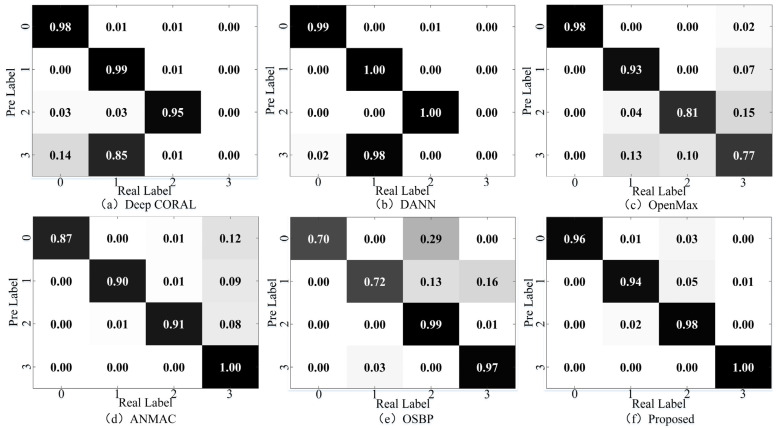
The confusion matrix of the results for transfer task a1. (**a**) Deep CORAL, (**b**) DANN, (**c**) OpenMax, (**d**) ANMAC, (**e**) OSBP, (**f**) Proposed.

Take the diagnosis task a1 in the laboratory gearbox dataset as an example, and randomly select some samples for feature visualization through the t-SNE algorithm, as shown in Figure 7. The confusion matrix of the diagnosis results is shown in Figure 8.

It can be found that the traditional domain adaptation methods DANN and Deep CORAL can well align each shared class in the feature extractor’s mapping space, and the recognition accuracy is close to 100%. However, the non-shared part in the target domain is confused with other classes, and the recognition accuracy is 0, as the models cannot recognize non-shared class samples. In OpenMax, the shared class samples can be well aligned, the diagnostic accuracy reaches more than 90%, and the samples can be well aligned in the feature space. However, the non-shared class samples have severe aliasing, reducing the overall accuracy. In OSBP, there is a tendency for non-shared samples to be separated. However, due to the settings of the fixed threshold, the model has a negative transfer problem, resulting in the wrong domain alignment of the shared class samples. ANMAC alleviates the problems in OSBP. The recognition accuracy of the shared samples is close to 90%, but there is still room for improvement. In the proposed method, the positive transfer between the shared classes is promoted by the weighting module. The results show that the shared classes can be aligned in the feature space, and the recognition accuracy is over 96%. Additionally, the non-shared samples can be separated well, the confusion with the shared class samples is significantly reduced, and the recognition accuracy is close to 100%, proving the proposed method’s advantages in cross-domain open set fault diagnosis.

### 4.6. Experimental Results and Analysis of the Different Equipment

The diagnostic accuracy of this method and the comparison method in different equipment is shown in Table 6. The average accuracy rates of DANN, CORAL, OpenMax, ANMAC, OSBP, and the proposed method in the two diagnostic tasks were 43%, 37.6%, 43.4%, 67%, 60.5% and 80.2%, respectively. Specifically, due to the significant differences of the signals collected by different equipment, the performances of traditional domain adaptation methods DANN and CORAL in open set fault diagnosis are abysmal, and due to the existence of non-shared samples, many samples are aliased in the feature space alignment, resulting in a low diagnostic accuracy. As a typical method for open set recognition, OpenMax has a significant decrease in recognition accuracy due to the large differences between the two domains. OSBP and ANMAC have some effects on the open set diagnosis of different equipment, but they cannot provide stable and efficient diagnosis results. The proposed method not only promotes the alignment of each class in the shared class and the separation of non-shared samples but also uses the differences between the two classifiers to improve the model’s generalization effectively. The average diagnostic accuracy of the task can reach about 80%, which is superior to all comparable models, providing the possibility of feature transfer between different equipment.

**Figure 9:**
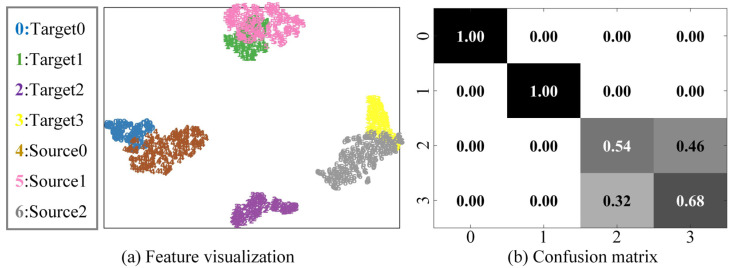
Results analysis of transfer task: IMS→CWRU. (**a**) Feature visualization, (**b**) Confusion matrix.

The feature visualization of the random sample and the confusion matrix of the diagnostic results in the transfer task IMS → CWRU are shown in Figure 9. Since the IMS is a life cycle dataset, the samples contain rich feature information, which makes the diagnosis accuracy of the IMS as the source domain relatively high. In the sharing part, most samples can accurately align the corresponding classes in the source domain, with an accuracy rate of about 85%. Only the sample in the health condition and the inner race fault sample as a non-shared class have a significant overlap, which provides feasibility for open set fault diagnosis between different equipment.

### 4.7. Test on Adaptability

All of the above experimental data are from laboratories or public datasets, the interference in the signal is minor, and the fault features are significant. However, in the actual industry, the signal collected by sensors often has large interference, such as noise and other sound sources. The dataset of the overhung impeller centrifugal pump is used to verify the robustness and generalization of the proposed method. The dataset is collected under industrial scenes, and the signal contains large interference. The equipment shown in Figure 4d selects the rolling fault as the non-shared class in the two domains. The transfer task c1 takes the data under 743 rpm working conditions as the source domain and the 743 rpm data in the transfer task c2 as the target domain.

**Figure 10:**
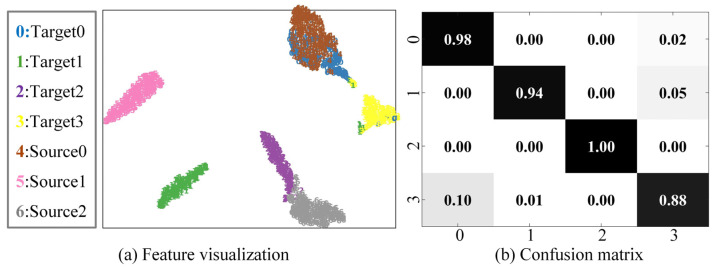
Results analysis of transfer task c1. (**a**) Feature visualization, (**b**) Confusion matrix.

The diagnostic results of all the methods in the two diagnosis tasks are shown in Table 7. The average accuracy of DANN, CORAL, OpenMax, ANMAC, OSBP, and the proposed method are 74.93%, 72.87%, 76.24%, 91.13%, 83.07%, and 96.07%, respectively. Obviously, due to the interference in the signal, the diagnostic accuracy of all the methods decreased to a certain extent, and from the standard deviation, the stability of the diagnosis fluctuated. The method proposed in this paper still achieved the highest diagnostic accuracy among all the diagnostic models, and the standard deviation was low, indicating that the method had good robustness and generalization. The feature visualization of the random sample and the confusion matrix of the diagnostic results in the transfer task c1 are shown in Figure 10. It can be seen that, in the feature space, a small number of samples have severe feature aliasing. Still, most of the samples can align well with the corresponding classes, indicating that the method can realize the transfer of fault features during large interference.

### 4.8. The Limitations and Scope

From the above experimental results, the proposed method performs well in the open set fault diagnosis of rotating machinery. To evaluate the efficiency of the proposed method, Table 8 lists the average training times and model parameters of all the methods under the same conditions. The training time is the average time required for each epoch, and the parameter count is the parameter size required for building the model. As can be seen from the results in the table, since the main network model of all the methods is the same, the difference between the parameter count and the parameter size is small. In terms of training time, the CORAL and OpenMax algorithms have advantages. Other algorithms spend more time in training due to the use of adversarial learning. For the proposed method, due to the calculation of the similarity between the class center and the samples in the feature space, the training takes the most time, which is the limitation of the proposed method and one of the future research directions.

Regarding the application scope, the CORAL and DANN algorithms play an excellent role in closed set fault diagnosis. However, neither of these two methods can recognize unknown samples. OpenMax, ANMAC, and OSBP can realize open set fault diagnosis, but the accuracy needs to be improved. The proposed method improves the recognition accuracy in open set fault diagnosis, and the time spent is worthwhile from the results.

## 5. Conclusions and Prospects

In this paper, a cross-domain open set fault diagnosis method based on WDADC was proposed. This method makes the time-frequency image of the signal as the input of model and extracts the features of the sample. Then, a weighting module is designed to assign larger weights to samples with a higher similarity, and a weighted cross-entropy loss function is constructed to promote a positive transfer between the classes of shared samples to achieve a continuous fault diagnosis. In addition, to improve the diagnostic effect of the model on different equipment, double classifiers are designed to enhance the generalization of the model. The experimental results showed that WDADC has advantages in open set fault diagnosis. Under different working conditions of the gearbox, the average accuracy of the proposed method is more than 95%, about 30% higher than the traditional domain adaptation method and over 10% higher than the typical method for open set recognition. More importantly, the experiment has verified the feasibility of the proposed method in feature transfer between different equipment. As shown in the results, the proposed method still has an average accuracy of more than 80% in the transfer tasks between the different equipment, which is 35%, 40%, 35%, 10%, and 15% higher than that of DANN, CORAL, OpenMax, ANMAC, and OSBP. This shows that the proposed method can be extended to different equipment fault diagnoses.

The proposed method achieves the open set fault diagnosis performance well in these datasets. However, in industrial practice, the collected datasets may have the problem of imbalance, which will significantly affect the diagnosis results of the model. This will be a future research direction.

## Figures and Tables

**Table 1:** The application of TL in fault diagnosis.

	Problems	Types	Papers
Transfer Learning for fault diagnosis	Closed set	Instance-based	[22,23]
		Mapping-based	[24,25]
		Model-based	[26,27]
		Adversary-based	[28,29,30,31]
	Open set	Discriminate-based	[32,33,34,35]
		Generation-based	[36,37]

**Table 2:** The transfer task settings in the lab gearbox dataset.

Task	Source	Target	Shared Class
a1	1800	1500	IR, IT, I
a2	1500	1800	IR, IT, I
a3	1800	1200	IR, OT, I
a4	1200	1800	IR, OT, I
a5	1500	1200	OT, IT, I
a6	1200	1500	OT, IT, I

**Table 3:** The transfer task settings in the CWRU dataset.

Task	Source	Target	Task	Source	Target
b1	0	1	b4	2	1
b2	1	0	b5	0	2
b3	1	2	b6	2	0

**Table 4:** Diagnostic results in the lab gearbox dataset (accuracy (%) ± standard deviation).

Task	DANN	CORAL	OpenMax	ANMAC	OSBP	Proposed
a1	66.31 ± 0.17	64.93 ± 0.33	85.67 ± 0.79	92.87 ± 0.35	85.62 ± 0.75	97.02 ± 0.34
a2	64.93 ± 0.51	61.60 ± 0.79	83.27 ± 0.83	90.13 ± 0.34	86.67 ± 0.31	92.33 ± 0.53
a3	66.18 ± 0.33	65.76 ± 0.42	80.80 ± 0.34	91.73 ± 0.26	86.76 ± 0.58	96.49 ± 0.21
a4	65.31 ± 0.38	63.44 ± 0.67	81.87 ± 0.51	94.07 ± 0.87	88.07 ± 0.69	97.16 ± 0.07
a5	66.35 ± 0.25	65.36 ± 0.43	83.27 ± 0.87	93.31 ± 0.09	82.71 ± 0.52	96.20 ± 0.13
a6	65.76 ± 0.23	64.96 ± 0.57	80.07 ± 0.21	91.93 ± 0.87	83.91 ± 0.39	96.87 ± 0.30
AVG	65.81	64.34	82.49	92.34	85.62	96.01

**Table 5:** Diagnosis results in the CWRU dataset (accuracy (%) ± standard deviation).

Task	DANN	CORAL	OpenMax	ANMAC	OSBP	Proposed
b1	75.00 ± 0	74.87 ± 0.32	90.15 ± 0.25	98.63 ± 0.23	89.80 ± 0.45	98.67 ± 0.26
b2	75.00 ± 0	74.33 ± 0.27	92.34 ± 0.18	98.13 ± 0.31	91.77 ± 0.37	98.67 ± 0.17
b3	75.00 ± 0	74.50 ± 0.08	89.57 ± 0.49	95.03 ± 0.77	89.43 ± 0.74	98.30 ± 0.22
b4	75.00 ± 0	75.00 ± 0	90.73 ± 0.39	98.07 ± 0.34	90.93 ± 0.23	98.83 ± 0.24
b5	75.00 ± 0	74.17 ± 0.41	93.70 ± 0.09	97.97 ± 0.37	88.93 ± 0.59	98.53 ± 0.11
b6	75.00 ± 0	74.17 ± 0.21	91.63 ± 0.12	97.63 ± 0.65	88.77 ± 0.47	98.20 ± 0.34
AVG	75.00	74.51	91.35	97.58	89.94	98.53

**Table 6:** Diagnosis results in the different equipment (accuracy (%) ± standard deviation).

Task	DANN	CORAL	OpenMax	ANMAC	OSBP	Proposed
IMS → CWRU	45.93 ± 2.30	38.33 ± 1.66	47.54 ± 3.03	69.87 ± 1.91	65.50 ± 1.30	80.53 ± 1.02
CWRU → IMS	40.03 ± 2.35	36.83 ± 1.85	39.33 ± 1.54	64.15 ± 1.33	58.40 ± 1.74	79.93 ± 0.91
AVG	42.98	37.58	43.44	67.01	60.45	80.23

**Table 7:** Diagnosis results in the centrifugal pump dataset (accuracy (%) ± standard deviation).

Task	DANN	CORAL	OpenMax	ANMAC	OSBP	Proposed
c1	75.00 ± 0	72.53 ± 1.08	72.87 ± 1.68	90.13 ± 1.79	82.67 ± 1.41	95.13 ± 0.88
c2	74.86 ± 0.32	73.2 ± 1.17	79.61 ± 3.94	92.13 ± 1.07	83.46 ± 0.81	97.00 ± 0.54
AVG	74.93	72.87	76.24	91.13	83.07	96.07

**Table 8:** The analysis of the complexity and computation time.

Method	Training Time (s)	Parameter Count	Params Size (MB)
DANN	3.49	660,742	2.52
CORAL	2.11	660,136	2.52
OpenMax	2.27	660,136	2.52
ANMAC	4.28	669,183	2.55
OSBP	2.79	660,540	2.52
Proposed	4.52	660,944	2.52

## Data Availability

Not applicable.

## Nomenclature

*C*	Classification module
*C_a_*	Class center of the a-th class
*D_i_*	Distance between the sample and class center
*D_JS_*	Jensen–Shannon divergence
*D_KL_*	Kullback–Leibler divergence
$F_{i}^{t}$	Features of the *i*-th target domain sample
$F_{i}^{s}$	Features of the *i*-th source domain sample
*G*	Feature extraction module
*L_CE_*	the cross-entropy loss
*L_d_*	the total loss
*L_s_*	Discrepancy loss of classifiers
$L_{t}^{i}$	the binary cross-entropy loss of the *i*-th target domain sample
*M*	Number of fault classes in the source domain
*n_s_*	Number of labeled samples
*n_t_*	Number of unlabeled samples
*n_a_*	Number of *a*-th class samples
$p_{x_{i}^{t}}^{M+1}$	Probability that the *i*-th sample is recognized as a non-shared class.
*p_c1_*	Probability distribution of classifier 1’s output
*p_c2_*	Probability distribution of classifier 2’s output
*t*	Threshold
*v*	Dimension of the Feature extraction module’s output
*W*	Weighting module
*w_i_*	Weight of the *i*-th sample
$x_{i}^{s}$	The *i*-th sample in the source domain
$x_{i}^{t}$	The *i*-th sample in the target domain
$y_{i}^{s}$	Label of the *i*-th sample in the source domain
$y_{i}^{t}$	Label of the *i*-th sample in the target domain
